# Searching for the Oldest Baobab of Madagascar: Radiocarbon Investigation of Large *Adansonia rubrostipa* Trees

**DOI:** 10.1371/journal.pone.0121170

**Published:** 2015-03-25

**Authors:** Adrian Patrut, Karl F. von Reden, Pascal Danthu, Jean-Michel Leong Pock-Tsy, Roxana T. Patrut, Daniel A. Lowy

**Affiliations:** 1 Babeş-Bolyai University, Faculty of Chemistry, Cluj-Napoca, Romania; 2 NOSAMS Facility, Department of Geology & Geophysics, Woods Hole Oceanographic Institution, Woods Hole, Massachusetts, United States of America; 3 Cirad, UPR BSEF, Montpellier, France; 4 DP Forêts et Biodiversité, Antananarivo, Madagascar; 5 Babeş-Bolyai University, Faculty of Biology and Geology, Cluj-Napoca, Romania; 6 Northern Virginia Community College (Nova University), Alexandria Campus, Alexandria, Virginia, United States of America; New York State Museum, UNITED STATES

## Abstract

We extended our research on the architecture, growth and age of trees belonging to the genus *Adansonia*, by starting to investigate large individuals of the most widespread Malagasy species. Our research also intends to identify the oldest baobabs of Madagascar. Here we present results of the radiocarbon investigation of the two most representative *Adansonia rubrostipa* (fony baobab) specimens, which are located in south-western Madagascar, in the Tsimanampetsotse National Park. We found that the fony baobab called “Grandmother” consists of 3 perfectly fused stems of different ages. The radiocarbon date of the oldest sample was found to be 1136 ± 16 BP. We estimated that the oldest part of this tree, which is mainly hollow, has an age close to 1,600 yr. This value is comparable to the age of the oldest *Adansonia digitata* (African baobab) specimens. By its age, the Grandmother is a major candidate for the oldest baobab of Madagascar. The second investigated specimen, called the “polygamous baobab”, consists of 6 partially fused stems of different ages. According to dating results, this fony baobab is 1,000 yr old. This research is the first investigation of the structure and age of Malagasy baobabs.

## Introduction

The genus *Adansonia* belonging to the Bombacoideae, a subfamily of Malvaceae, consists of nine species out of which six are endemic to Madagascar [1–3].

Up to present, the research dedicated to the architecture, growth and age of *Adansonia* trees was performed exclusively on the African baobab (*Adansonia digitata* L.). We found that all large African baobabs are multi-stemmed and exhibit preferentially ring-shaped structures; the oldest dated specimens were found to be between 1,000 and 2,000 years old [4–10]. We extended our research on the *Adansonia* genus by starting to investigate large individuals of the most representative Malagasy species, *i*.*e*., *Adansonia rubrostipa* Jum. & H. Perrier (fony baobab), *Adansonia za* Baill. (za baobab) and *Adansonia grandidieri* Baill. (Grandidier’s baobab or reniala), which grow in west and south; each of these species is represented by over 1 million individuals [2,11,12]. The research is based on our approach which consists of AMS (accelerator mass spectrometry) radiocarbon dating of small wood samples collected from different areas of the trunk/stems of large baobabs [6,8]. Our research also intends to identify the oldest baobabs of Madagascar and to answer two main questions: (i) are there Malagasy baobabs over 1,000 years old? (ii) if yes, which species do these individuals belong to?

**Fig 1:**
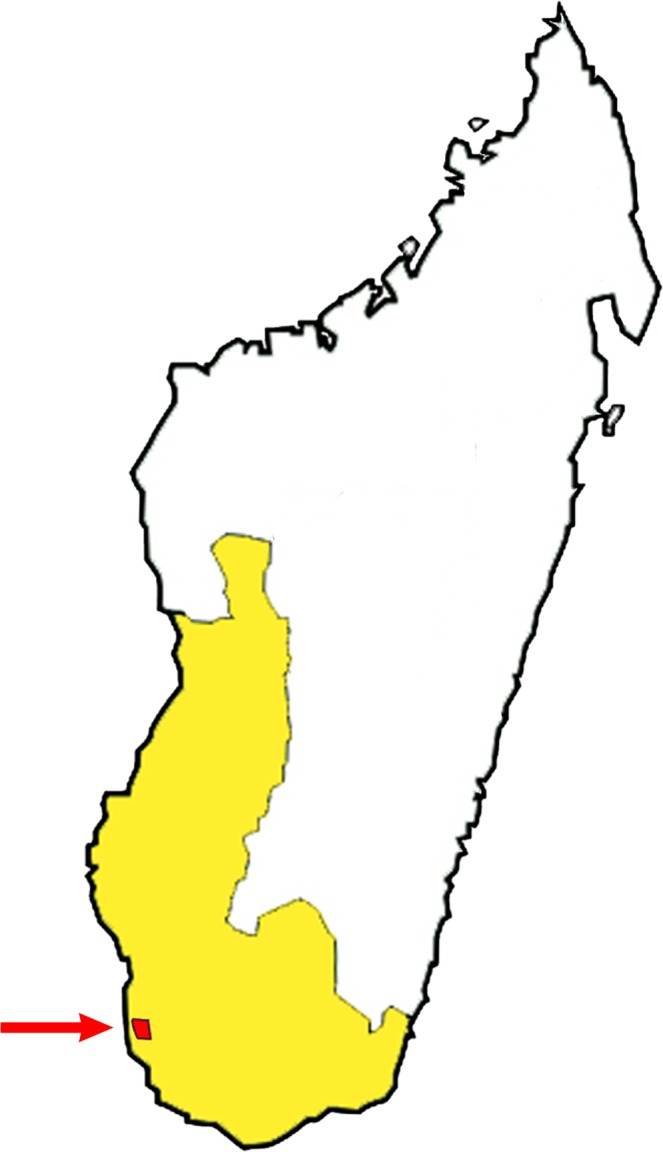
Map of Madagascar, showing the Toliara province (in yellow) and the position of the Tsimanampetsotse National Park (marked by the red arrow).

*A*. *rubrostipa* is represented by small to medium and also by some relatively large, rather bizarre-looking trees. The fony baobabs have bottle-shaped or quasi-cylindrical trunks and short horizontal branches that turn upwards at the ends [2]. According to recent estimates, there are up to 1.5 million *A*. *rubrostipa* individuals which are widespread along the west coast of Madagascar. The largest known and probably the oldest fony baobabs are both located in the Tsimanampetsotse National Park, Toliara Province (Fig. 1). Here we present the radiocarbon investigation results of these two most representative *A*. *rubrostipa* individuals.

## Materials and Methods

### Ethics statement

The investigation and sampling of the baobabs was approved and authorised by the Forestry Direction of the Ministry of Environment, Ecology and Forestry of Madagascar and by the Madagascar National Parks. The Madagascar National Parks, Tsimanampetsotse National Park and PARRUR project (ECOBAO) provided scientific assistance and support in the investigation. The baobabs were not endangered by the sampling. After each coring, the increment borer was disinfected with methyl alcohol. The small coring holes were sealed with Steriseal (Efekto), a special polymer sealing product, for preventing any possible infection of the trees.

### The two baobabs and their area

The two fony baobabs are located in the Tsimanampetsotse National Park, at a distance of only 1.5 km apart from each other. The mean annual rainfall in this very arid area is around 350 mm.

**Fig 2:**
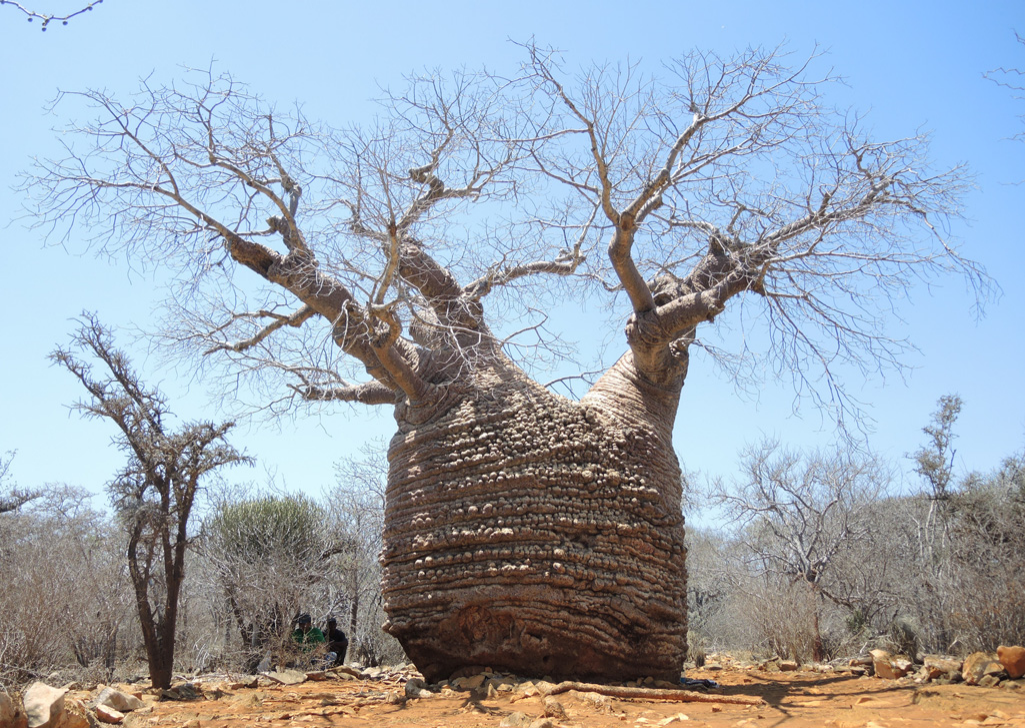
General view of the old Grandmother.

**Fig 3:**
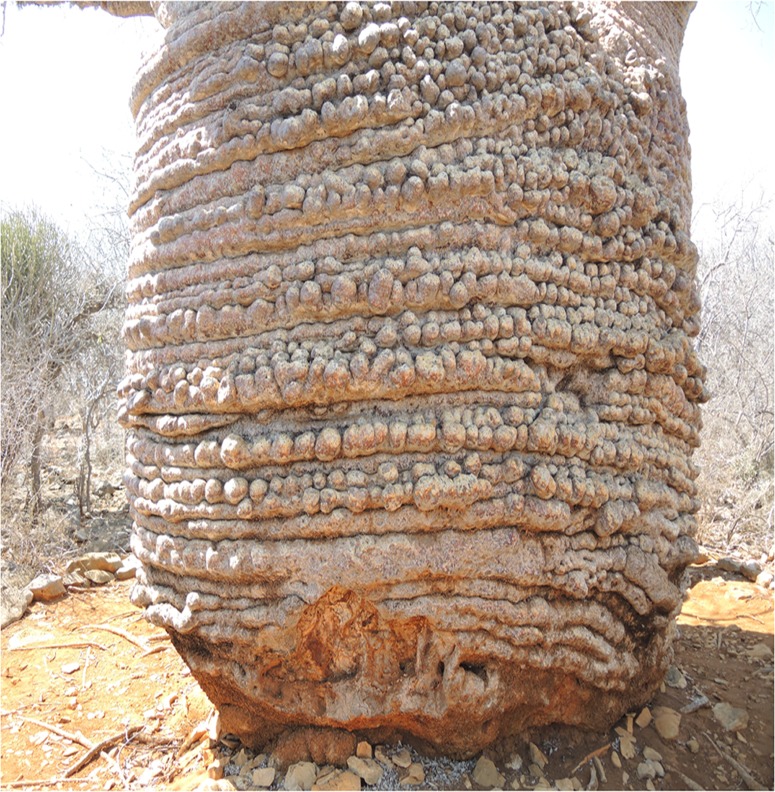
The strange looking tri-stemmed trunk of the Grandmother showing its various sized bulbuous formations.

**Fig 4:**
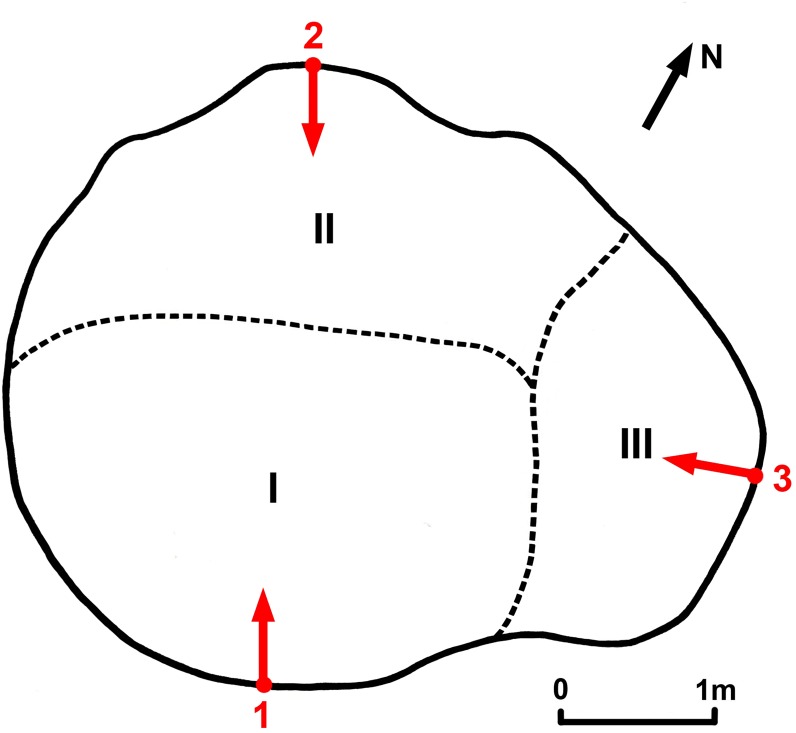
Cross-section of Grandmother (at 1.30 m above ground) showing the 3 fused stems (I, II and III) and the projection of sampling points’ positions (1, 2 and 3).

The”Grandmother” (La grand-mère) is generally considered the oldest *A*. *rubrostipa*. Its GPS coordinates are 24°02.707' S, 043°45.266' E and the altitude is 36 m. It has a height of 7.47 m and a circumference at breast height (cbh) of 9.67 m. The overall wood volume is 25 m^3^. The tree, which has a pot shape, consists of 3 perfectly fused stems (Figs. 2–4). However, some fusion lines of stems can be identified at the upper part of the trunk.

**Fig 5:**
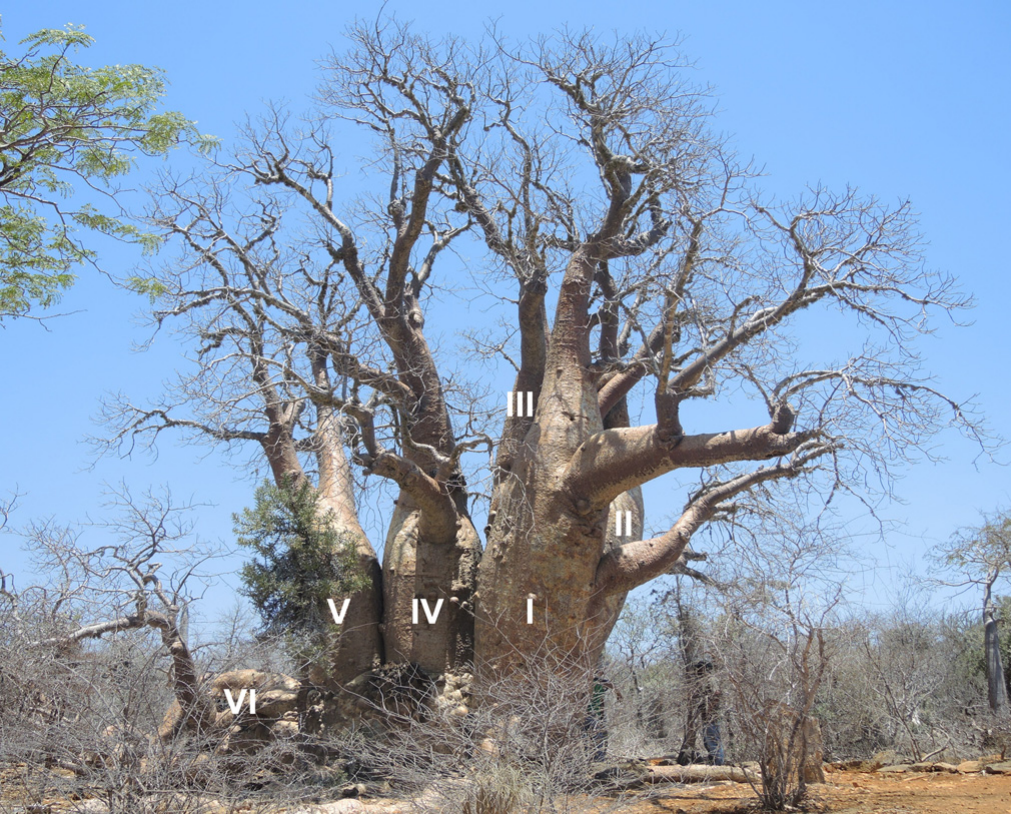
Western view of the polygamous baobab with stem numbering (from I to VI).

**Fig 6:**
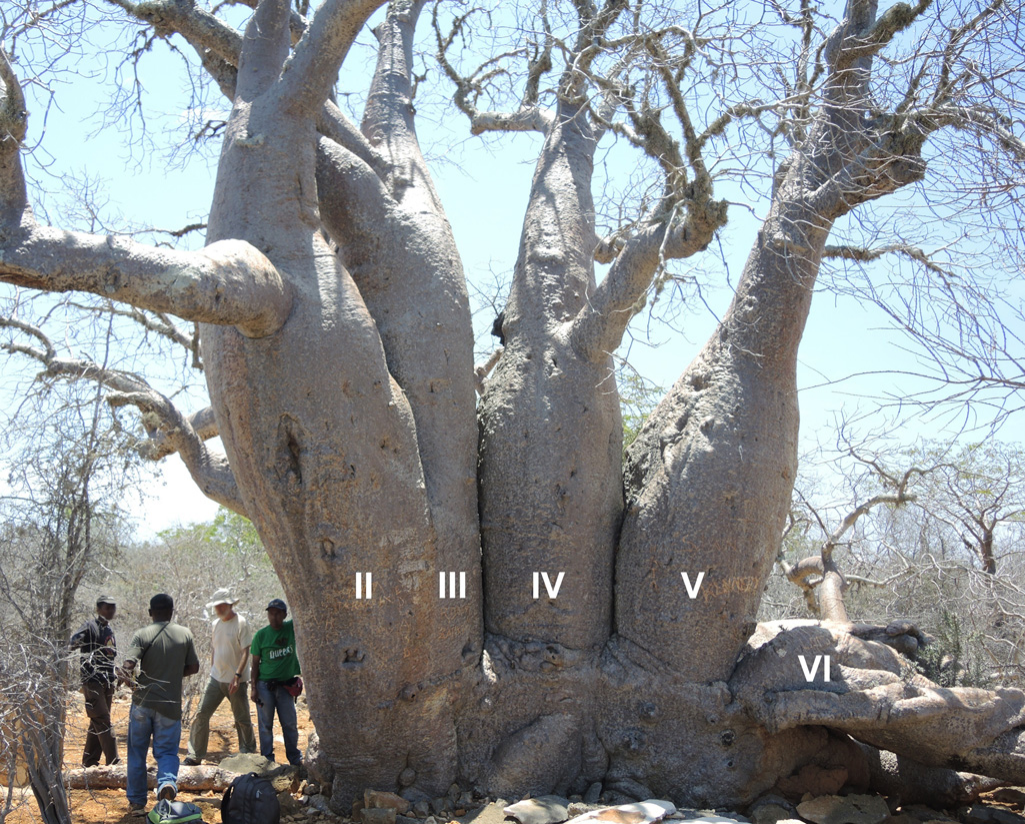
Eastern view of the polygamous baobab with stem numbering.

The ”polygamous baobab” (Le baobab polygame), the largest known *A*. *rubrostipa*, is located at 24°03.060' S, 043°45.418' E and the altitude is 27 m. It has a maximum height of 14.2 m and a restored circumference (cbh) of 13.50 m. The overall wood volume is 60 m^3^. It exhibits a cluster structure with 6 partially fused stems. The stem from the northern extremity collapsed some time ago, but it is still alive (Figs. 5 and 6).

### Sample collection

Several wood samples were collected from the outer part of the 3 stems of Grandmother, at convenient heights between 1.27–1.55 m. The longest samples from each stem (labelled GM-1, GM-2, GM-3) were investigated. The stem numbering (I, II, III) and the sampling positions of the longest samples are shown in Fig. 4.

Additional 5 wood samples (labelled PB-1 to PB-5) were collected from 5 stems of the polygamous baobab, at heights between 1.30–1.53 m. The stem numbering (from I to VI) is displayed in Figs. 5 and 6.

The samples were collected by using Haglöf increment borers (0.80–1.00 m long, 0.010–0.012 m inner diametre). Several small pieces/segments of the length of 0.001 m were extracted from determined positions of the 10 samples and investigated by AMS radiocarbon dating.

### Sample preparation

The standard acid-base-acid pretreatment method [13] was used for removing soluble and mobile organic components. The resulting samples were combusted to CO_2_ *via* the closed tube combustion method [14]. Next, CO_2_ was reduced to graphite on iron catalyst, under hydrogen atmosphere [15]. The graphite samples were analysed by AMS.

### AMS measurements

AMS radiocarbon measurements were performed at the NOSAMS Facility of the Woods Hole Oceanographic Institution by using the Pelletron Tandem 500 kV AMS system [16,17]. The obtained fraction modern values were ultimately converted to a radiocarbon date.

### Calibration

Fraction modern values were calibrated and converted into calendar ages with the OxCal v4.2 for Windows [18], by using the SHCal13 atmospheric data set [19].

## Results

### AMS results and calibrated ages

Fraction modern values and radiocarbon dates bp (before present, referring to ad 1950) of 10 wood samples/segments which originate from the Grandmother (GM) and the polygamous baobab (PB) are displayed in Tables 1 and 2. Radiocarbon dates and errors were rounded to the nearest year. The accession numbers in the NOSAMS database of the 10 radiocarbon analyses are also listed in Tables 1 and 2.

**Table 1:** AMS Radiocarbon dating results and calibrated calendar ages of samples collected from the Grandmother.

Sample (Segment)	Depth in the wood^1^ [Distance to stem pith^1^] (10^-2^ m)	Fraction modern [error]	Radiocarbon date [error] (^14^C yr bp)	Cal ad range (1σ or 2σ)^2^ [confidence interval]	Sample age [error] (yr in ad 2014)	NOSAMS Accession #
GM-1(a)	0.5 [115]	0.9765 [±0.0026]	191 [±21]	1666–1712 [25.8%] **1718–1814 [49.7%]** 1836–1884 [12.7%] 1924-… [7.3%]	250 [±50]	OS-110648
GM-1(b)	20 [115]	0.9189 [±0.0032]	679 [±26]	1300–1320 [25.6%] **1344–1388 [42.6%]**	650 [±20]	OS-108121
GM-1(c)	46 [115]	0.8681 [±0.0020]	1136 [±16]	**892–995 [93.7%]** 1005–1013 [1.7%]	1070 [±50]	OS-112137
GM-2	51 [80]	0.9074 [±0.0020]	781 [±16]	**1268–1288 [68.2%]**	735 [±10]	OS-111763
GM-3	47 [65]	0.9492 [±0.0026]	419 [±21]	**1457–1498 [58.2%**] 1599–1608 [10.0%]	535 [±20]	OS-110647

^1^from the sampling point

^2^with the SHCal13 calibration

**Table 2:** AMS Radiocarbon dating results and calibrated calendar ages of samples collected from the polygamous baobab.

Sample (Segment)	Depth in the wood^1^ [Distance to stem pith^1^] (10^-2^ m)	Fraction modern [error]	Radiocarbon date [error] (^14^C yr bp)	Cal ad range (1σ)^2^ [confidence interval]	Sample age [error] (yr in ad 2014)	NOSAMS Accession #
PB-1	55 [82]	0.8994 [±0.0034]	852 [±28]	**1214–1266 [68.2%]**	785 [±25]	OS-110646
PB-2	47 [75]	0.9420 [±0.0025]	480 [±20]	**1440–1456 [68.2%]**	565 [±10]	OS-108826
PB-3	39 [60]	0.9463 [±0.0029]	443 [±23]	**1448–1487 [68.2%]**	545 [±20]	OS-110644
PB-4	41 [65]	0.9602 [±0.0030]	326 [±24]	**1512–1549 [34.9%]** 1560–1572 [8.3%] 1622–1646 [25.1%]	485 [±20]	OS-108824
PB-5	40 [65]	0.9621 [±0.0029]	310 [±23]	1514–1543 [25.3%] **1624–1654 [42.9%]**	375 [±15]	OS-109280

^1^from the sampling point

^2^with the SHCal13 calibration

The 1σ probability distribution (68.2%) was typically selected to derive calibrated age ranges. For 4 sample segments, the 1σ distribution is consistent with one range of calendar years, while for other 4 segments, the 1σ distribution corresponds to two or three ranges of calendar years. For these segments, the confidence interval of one range is considerably greater than that of the others; therefore, it was selected as the cal ad range of the sample for the purpose of this discussion. For 2 sample segments, there are two 1σ ranges with close confidence intervals. In these special cases *i*.*e*., GM-1(a) and GM-1(c), we used the higher 2σ probability distribution (95.4%) for calibration.

For obtaining single age values, we derived a mean calibrated (cal) age of each segment from the selected range (marked in bold). Calibrated ages of samples/segments represent the difference between ad 2014 and the mean ad value of the selected range, with the corresponding error. All calibrated sample/segment ages, as well as stem ages and tree ages are expressed in (calendar) yr in ad 2014. Calibrated ages and errors were rounded to the nearest 5 yr.

### Results of samples collected from the Grandmother

Even if the borer penetration in the soft wood was quasi-complete in all cases, the collected samples were much shorter. This shows that more than half of the tree is hollow, due to decay. We extracted and dated 3 segments (labelled a, b, c) from sample GM-1, which originate from the largest stem I. The innermost and oldest segment GM-1(c) corresponds to the sample end located at 0.46 m from the sampling point. Its radiocarbon date was found to be 1136 ± 16 bp, which corresponds to a calibrated age of 1070 ± 50 yr. The second oldest dated segment GM-2(b), which corresponds to a depth of 0.20 m in the wood, has an age of 650 yr (neglecting the error). The ages and distances of segments GM-1(b) and GM-1(c) indicate that stem I grew 0.26 m in 420 yr in the sampling direction. This value indicates a mean radial increase of 0.62 x 10^-3^ m yr^-1^ over the time frame ad 945–1365.

The outermost segment GM-1(a), which was adjacent to the bark, is not as young as it would be expected. Its calibrated age of 250 yr shows that the large stem I stopped growing around ad 1765. One should, however, mention that the calibration of such low radiocarbon dates is very difficult and also somewhat uncertain, particularly with the southern data sets. According to ages and distances of segments GM-1(a) and GM-1(b), stem I grew 0.20 m in 400 yr in the sampling direction. The value indicates a slightly lower mean radial increase, of 0.50 x 10^-3^ m yr^-1^ over the time frame ad 1365–1765.

We dated the deepest segments from samples GM-2 and GM-3, which were collected from the second stem II and from the smallest stem III. These segments correpond to a depth in the wood of 0.52 and 0.55 m and have ages of 735 ± 10 and 535 ± 20 yr.

### Results of samples collected from the polygamous baobab

The 5 samples PB-1, PB-2, PB-3, PB-4 and PB-5 originate from the five standing stems, *i*.*e*., I, II, III, IV and V. The 5 samples were also shorter than the penetration distance of the increment borer, showing the presence of significant hollow parts in each stem. In all cases, we extracted and dated the innermost/ deepest segment of each sample.

The oldest dated segment PB-1 originates from stem I, at a distance of 0.52 m from the sampling point. Its radiocarbon date of 852 ± 28 bp corresponds to a calibrated age of 785 ± 25 yr. The other 4 sample segments exhibit a continuous age decrease from PB-2 to PB-5 having ages between 565 and 375 yr.

## Discussion

### Ages of Grandmother and its stems

For detemining the true age of Grandmother, which is identical to the age of stem I, we used the age of the oldest segment GM-1(a), the estimated distance to the calculated pith of stem I from the sampling point (1.15 m) and from the oldest segment (0.69 m) and also the mean growth rates for 650 and 420 yr (see Table 1 and Fig. 4). In a conservative estimate, the age of stem I and, consequently, of Grandmother is 1,500–1,700 yr, *i*.*e*., 1,600 ± 100 yr. According to this value, the Grandmother started growing around ad 400.

The ages of stems II and III were determined from the ages of samples GM-2 and GM-3 and the distances from these sample ends to the presumptive piths of stems II and III. According to these values, the estimated ages of stems II and III are of ca. 1,100 and 750 yr.

### Ages of polygamous baobab and its stems

The age of the polygamous baobab, which is identical to the age of its oldest stem I, was determined by extrapolating the calibrated age of sample PB-1 to the corresponding pith (see Table 2). Therefore, we consider that the polygamous baobab and its stem I are 1,000 ± 100 yr old. The ages of the other 4 sampled stems were estimated in a similar manner. Thus, stem II and stem III, which are fused completely up to a height of 2.60 m, are both around 750 yr old. Stems IV and V have estimated ages of 600 and 500 yr. The fallen stem VI, which broke partially in the collapse, was not sampled. According to its size, we estimate that it is around 300 yr old.

### Architecture and growth of the two fony baobabs

The two large and old *A*. *rubrostipa* specimens started growing as single-stemmed and became over time multi-stemmed, thus developing different shapes. This evolution is due to the general ability of baobabs, which we identified for the first time for *A*. *digitata* [5,9,10], to produce stems during their life cycle, such as other trees produce branches.

The Grandmother started growing its main stem I around ad 400. The tree produced new stems in ad 900 and 1250, developing over time its well-known pot shape with 3 perfectly fused stems. The main stem I stopped growing in ad 1750, because of old age.

The main stem I of the polygamous baobab started growing around ad 1000. Stems II and III emerged probably together in ad 1250 and the fony baobab gained a triangular structure. Next, 3 new stems emerged succesively toward north in ad 1400, 1500 and 1700. Thus, the polygamous baobab developed its cluster structure, with 6 very distinct bottle-shaped stems, which are fused only in the proximity of their base and have separate canopies.

## Conclusions

We investigated by AMS radiocarbon dating two representative *Adansonia rubrostipa* specimens, which are located in a very arid area of the Tsimanampetsotse National Park, in south-western Madagascar. According to our research, the fony baobab called Grandmother, which has a typical pot shape, consists of 3 fused stems of different ages. The radiocarbon date of the oldest collected wood sample was of 1136 ± 16 bp, which corresponds to a calibrated age of 1070 ± 50 yr. We determined that the oldest stem of this baobab is 1,600 ± 100 yr old. This value is close to the age of the oldest dated *Adansonia digitata* specimens. The other two stems of Grandmother exhibit ages of 1,100 and 750 yr. The second investigated specimen, the polygamous baobab, is composed of 6 partially fused stems. Its 6 stems have ages between 300 and 1,000 yr.

According to accurate dating results, the fony baobab becomes the second *Adansonia* species with individuals that can live over 1,000 yr. On the other hand, by its age, the Grandmother is a major candidate for being the oldest baobab of Madagascar.

This research is the first investigation of the architecture, growth and age of Malagasy baobabs.
